# Communicating about COVID-19 vaccine development and safety

**DOI:** 10.1371/journal.pone.0272426

**Published:** 2022-08-05

**Authors:** Alistair Thorpe, Angela Fagerlin, Jorie Butler, Vanessa Stevens, Frank A. Drews, Holly Shoemaker, Marian S. Riddoch, Laura D. Scherer

**Affiliations:** 1 Spencer Fox Eccles School of Medicine at University of Utah, Salt Lake City, UT, United States of America; 2 Salt Lake City VA Informatics Decision-Enhancement and Analytic Sciences (IDEAS) Center for Innovation, Salt Lake City, UT, United States of America; 3 Geriatrics Research, Education, and Clinical Center (GRECC), VA Salt Lake City Health Care System, Salt Lake City, UT, United States of America; 4 University of Utah College of Social and Behavioral Science, Salt Lake City, UT, United States of America; 5 University of Colorado School of Medicine, Aurora, CO, United States of America; 6 VA Denver Center for Innovation, Denver, CO, United States of America; Boston Children’s Hospital, UNITED STATES

## Abstract

**Purpose:**

Beliefs that the risks from a COVID-19 vaccine outweigh the risks from getting COVID-19 and concerns that the vaccine development process was rushed and lacking rigor have been identified as important drivers of hesitancy and refusal to get a COVID-19 vaccine. We tested whether messages designed to address these beliefs and concerns might promote intentions to get a COVID-19 vaccine.

**Method:**

We conducted an online survey fielded between March 8–23, 2021 with US Veteran (*n* = 688) and non-Veteran (*n* = 387) respondents. In a between-subjects experiment, respondents were randomly assigned to a control group (with no message) or to read one of two intervention messages: 1. a fact-box styled message comparing the risks of getting COVID-19 compared to the vaccine, and 2. a timeline styled message describing the development process of the COVID-19 mRNA vaccines.

**Results:**

Most respondents (60%) wanted a COVID-19 vaccine. However, 17% expressed hesitancy and 23% did not want to get a COVID-19 vaccine. The fact-box styled message and the timeline message did not significantly improve vaccination intentions, *F*(2,358) = 0.86, *p* = .425, $\eta_{P}^{2}$ = .005, or reduce the time respondents wanted to wait before getting vaccinated, *F*(2,306) = 0.79, *p* = .453, $\eta_{P}^{2}$ = .005, compared to no messages.

**Discussion:**

In this experimental study, we did not find that providing messages about vaccine risks and the development process had an impact on respondents’ vaccine intentions. Further research is needed to identify how to effectively address concerns about the risks associated with COVID-19 vaccines and the development process and to understand additional factors that influence vaccine intentions.

## Introduction

All currently available COVID-19 vaccines have demonstrated high efficacy against COVID-19 [1]. Maximizing the public health benefits from these vaccines depends on achieving high levels of vaccine coverage. One of the major barriers to achieving widespread coverage of COVID-19 vaccines is public hesitancy and reluctance to receive them [2]. Prior research has established that attitudes towards vaccines and intentions to receive or refuse them are driven by a multitude of factors [3]. Within this literature, there is consistent evidence that people who are hesitant or reluctant to receive a vaccine often have doubts about the benefits of vaccines and concerns about their general safety [4].

Throughout the pandemic, how people perceive the threat posed by COVID-19 has been shown to vary based on a number of factors such as age [5], political ideology [6, 7], and philosophical beliefs [8]. How people perceive the risk of COVID-19 matters as those who do not identify COVID-19 to be a serious threat are likely to undervalue the benefits of vaccination [4] and are therefore less likely to choose to receive a vaccine [9–12]. Furthermore, people who do not consider COVID-19 to present a serious threat may also be reluctant to receive a vaccine because they are more likely to believe that they are more at risk from potential harms from vaccination than from acquiring COVID-19 [4, 9, 10].

The perceived safety of a vaccine is another factor that can have a strong influence on peoples’ decisions about whether or not to get vaccinated [4, 13]. Current evidence suggests that people who are hesitant about receiving a COVID-19 vaccine are often concerned about the safety of the COVID-19 vaccine development process [14]. Producing a number of highly effective vaccines within a year of an infectious disease outbreak is undoubtedly one of humanity’s greatest medical achievements. It is important to acknowledge that this achievement was only possible because of decades of prior research and clinical trials on vaccine technology (e.g., mRNA and viral vectors) and on the public health response to coronaviruses following outbreaks of SARS and MERS [15, 16]. Unfortunately, the importance and rigor of this prior research has often been overlooked in public discourse, which has placed greater emphasis on the speed of the vaccine development process. As a consequence, concerns that the safety of the vaccines was compromised by rushed development or the use of experimental/untested technology have emerged [14]. High-profile media coverage of very rare side-effects following COVID-19 vaccination (e.g., anaphylaxis and thrombosis with thrombocytopenia syndrome) may have compounded this issue by disproportionately raising public concern about vaccine safety and further fueling public hesitancy [17, 18]. Relatedly, the emergency use authorization given to the COVID-19 vaccines likely also raised concerns about the safety of the vaccines and, in turn, increased public hesitancy [19, 20].

It is critical to find communication strategies to address the public’s concerns about the safety and development process of COVID-19 vaccines. Providing succinct visual communications designed to directly address these frequently cited reasons for COVID-19 vaccine hesitancy may be an effective method for reducing concerns about the vaccines and encouraging uptake. Indeed, prior research has shown that using graphics to visually communicate public health information has numerous potential benefits including improving understanding about health information [21, 22] and motivating engagement in protective health behaviors [23]. In addition, short visual communications are conducive to rapid and widespread dissemination, which can further amplify their impact at low cost [24]. In the hope of achieving these benefits, government and health organizations have been swift in developing short, visual, informative messages about the COVID-19 vaccines and in disseminating them on their websites (e.g., https://www.cdc.gov/coronavirus/2019-ncov/downloads/vaccines/COVID-19-mRNA-infographic_G_508.pdf) and social media accounts. However, as few studies have sought to test the efficacy of short visual communications designed to directly address frequently cited concerns about the COVID-19 vaccines, doing so represents a significant contribution to the literature and future public health interventions. In order to examine the effectiveness of these types of communications, in the present study we tested whether messaging about the comparative risks of getting COVID-19 vs. the risk of COVID-19 vaccines, and also messaging about the timeline of mRNA vaccine development, might promote intentions to get a COVID-19 vaccine.

## Materials and method

### Study population and recruitment

Respondents were recruited by Qualtrics Online Panels between March 8–23, 2021. This experiment was conducted in the third wave of a three-wave longitudinal study [25]. The study was administered online (in English) and was approved (deemed exempt) by the IRBs at the University of Utah and the Salt Lake City VA (Veterans Experiences During the COVID-19 Pandemic: IRB_00133198). Respondents consented to participate in this voluntary study and were compensated for their participation based on the terms of their panel agreement.

### Procedure

**Fig 1:**
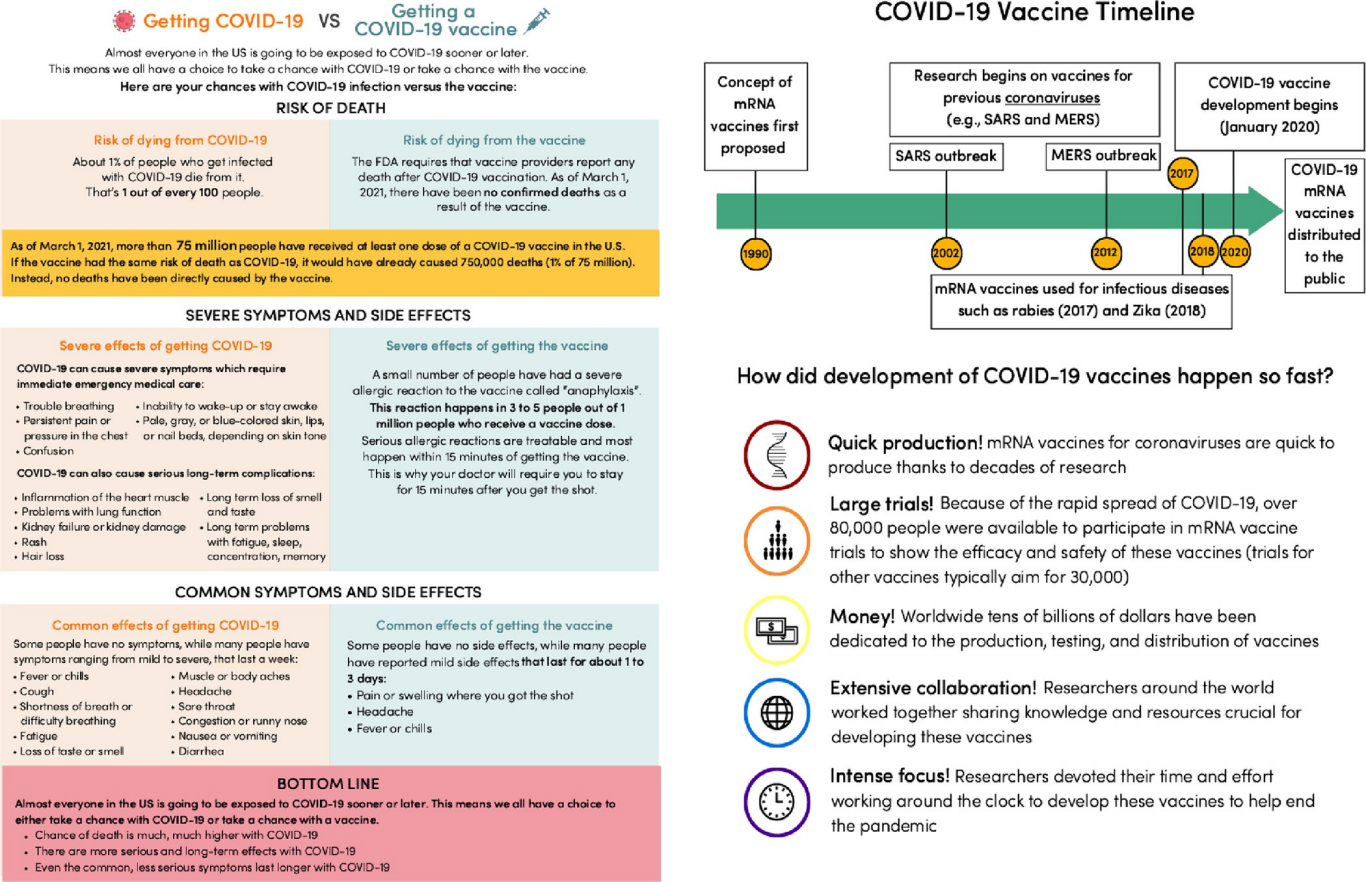
Factbox style message (left) about the risks of getting a vaccine compared to getting COVID-19 and the Timeline message (right) about the development of COVID-19 vaccines.

In a fully between-subjects experiment, respondents saw either a Schwartz et al. [26] factbox style message about the risks of getting a vaccine compared to getting COVID-19 (factbox message), a message about the development of COVID-19 vaccines (timeline message; both messages are presented in Fig 1), or no message (control group).

Both the factbox and timelines messages were developed by the study team as infographics displayed on a single page. As well as reflecting concerns about the COVID-19 vaccines reported in the existing literature, the messages were also designed to address the concerns reported by respondents in the first survey of the three-wave study this experiment was embedded in. Specifically, in the first wave survey we found that concerns that the risks of getting the vaccine outweighed the risks of getting COVID-19 (46%) and about the vaccine development process (21%) were reported as the most important reasons for not getting a COVID-19 vaccine among these respondents [27].

The factbox style message was chosen to convey information about the potential risks associated with getting COVID-19 compared with getting vaccinated as prior research suggests this format can improve comprehension and recall [28–30].

The timeline message was chosen in order to better understand whether these visualizations have an impact on attitudes towards vaccines, given their widespread use throughout the pandemic in academic outlets [15], in the mainstream media (https://www.nytimes.com/interactive/2020/04/30/opinion/coronavirus-covid-vaccine.html), and on social media (https://twitter.com/moderna_tx/status/1258370971855175680).

Respondent assignment to study conditions (no message [control], factbox message, timeline message) was carried out using the built-in randomizer function in Qualtrics’ survey flow.

Respondents first reported their COVID-19 vaccine intentions (“When a coronavirus vaccine becomes available to you, how interested are you in getting the vaccine?”) using a 5-point scale (1 = “I definitely do NOT want the vaccine”, 2 = “I do NOT want to get the vaccine”, 3 = “Unsure”, 4 = “I WANT to get the vaccine”, 5 = “I definitely WANT the vaccine”) and how long they intended to wait before getting vaccinated (“How soon after the COVID-19 vaccine becomes available to you would you become vaccinated?”) using an 8-point scale (1 = “Immediately”, 2 = “Less than one month”, 3 = “One month to less than 3 months”, 4 = “3 months to less than six months”, 5 = “6 months to less than 1 year”, 6 = “1 year to less than 2 two years”, 7 = “I would wait 2 years or more”, 8 = “I would never get it”).

We also asked respondents about their views regarding COVID-19 vaccine safety (“In your view, how safe is the COVID-19 vaccine?”) using a 5-point scale (1 = “Not at all safe”, 2 = “Slightly safe”, 3 = “Somewhat safe”, 4 = “Moderately safe”, 5 = “Extremely safe”) and the possibility of experiencing side effects (“How worried are you about experiencing side effects from the COVID-19 vaccine?”) using a 5-point scale (1 = “Not at all concerned”, 2 = “Slightly concerned”, 3 = “Somewhat concerned”, 4 = “Moderately concerned”, 5 = “Extremely concerned”).

### Pre-registered analyses

We ran two omnibus one-way ANOVA analyses to examine whether the COVID-19 vaccine risk comparison factbox message and vaccine development timeline message influenced 1) respondents’ intentions to get vaccinated and 2) the time they would wait to get vaccinated. Analyses were repeated controlling for age and gender.

We ran two additional omnibus one-way ANOVAs to examine whether 1) the factbox message comparing the risks associated with getting COVID-19 to the risks associated with receiving a COVID-19 vaccine reduced respondents’ concern about experiencing side effects from the COVID-19 vaccine and 2) whether the timeline message regarding the vaccine development process increased respondents’ views about how safe the COVID-19 vaccine is. All analyses were performed using RStudio statistical software Version 1.4.1106 [31].

### Exploratory analyses

These analyses were not planned a-priori, but were conducted after the unexpected finding that neither of the messages had a significant impact on respondents’ vaccination intentions or the time they wanted to wait before getting vaccinated.

We conducted exploratory analyses using omnibus one-way ANOVAs to examine the influence of the messages on vaccine intentions and time to getting vaccinated on two subsets of the total sample.

To examine the possibility that any impact of the messages on vaccination intentions could be dependent on whether respondents took the time to read and process the information provided in them, we re-ran our pre-registered analyses on a subset of respondents who spent at least 10 seconds on the page displaying the intervention messages.

Another possibility is that any impact of the messages on vaccination intentions may be limited only to respondents who were initially hesitant about getting a COVID-19 vaccine [32]. That is, an effect of the intervention might be dampened by ceiling effects on vaccination intentions. Thus, for the second subset we selected respondents who had reported that they were hesitant about getting a COVID-19 vaccine in an earlier survey fielded in December 2020 (i.e., reported that they either “do NOT want to get the vaccine” or “definitely do NOT want to get the vaccine”).

In light of the null effect of the information-based messages, we sought to identify which variables were associated with respondents’ vaccine intentions. To do so, we ran two multiple regression models: one to predict respondents’ vaccine intentions and one to predict how long they intended to wait before getting vaccinated. Predictor variables were the same in both models and included respondents’ age (≤65, 65 to 74, ≥75), Veteran status (Veteran or not), total comorbidities [33], health literacy [34], numeracy [35, 36], Race/Ethnicity (Non-Hispanic white or not), worry about getting COVID-19, COVID-19 risk perceptions, Emory Vaccine Confidence Index [37], beliefs about the importance of flu and COVID-19 vaccines, trust in health care [38], disbelief in science [39], beliefs in COVID-19 conspiracy theories [40], political beliefs, and medical maximizing [41].

## Results

A total of 1075 respondents completed the third wave of the study, of which 688 (64%) were United States Veterans. Median age of the sample was between 55 and 74 years old; 841 respondents (78%) were male, 819 respondents (76%) were non-Hispanic White, and the median household income was $70,000-$99,999. Sample retention from the first wave was 52% overall, 65% for Veteran and 38% for non-Veterans. Characteristics of respondents who completed only the first wave and those who completed all three are available on the project repository [25]. There were 361 (33%) respondents who reported having not received any vaccine doses, 243 (23%) who reported having received 1 dose, and 471 (44%) who reported having received 2 doses. As we were only interested in the effect of the interventions on respondents who had not yet been vaccinated, any respondents who reported having received at least 1 dose of a COVID-19 vaccine were excluded from the experimental portion of the survey, leaving a final sample of 361 respondents (Table 1).

**Table 1:** Respondent characteristics overall and according to group assignment.

		Overall	Control	Factbox	Timeline
		(N = 361)	(N = 120)	(N = 122)	(N = 119)
Age–*No*. *(%)*					
	18 to 34	46 (13)	15 (13)	15 (12)	16 (13)
	35 to 54	80 (22)	19 (16)	35 (29)	26 (22)
	55 to 74	205 (57)	75 (63)	64 (53)	66 (56)
	75 or older	28 (8)	10 (8)	8 (7)	10 (8)
	Did not respond	2 (1)	1 (1)	-	1 (1)
Gender–*No*. *(%)*					
	Female	123 (34)	41 (34)	43 (35)	39 (33)
	Male	235 (65)	79 (66)	77 (63)	79 (66)
	Non-binary/Third gender or Transgender man/Transman	3 (1)	-	2 (2)	1 (1)
Race/Ethnicity–*No*. *(%)*					
	Non-Hispanic White	252 (70)	86 (72)	78 (64)	88 (74)
	Non-Hispanic Black	49 (14)	17 (14)	15 (12)	17 (14)
	Hispanic	32 (9)	9 (8)	14 (11)	9 (8)
	Asian/Asian American	15 (4)	4 (3)	8 (7)	3 (3)
	American Indian/Alaskan Native	2 (1)	-	2 (2)	-
	Another race	7 (2)	3 (3)	4 (3)	-
	Multiracial	4 (1)	1 (1)	1 (1)	2 (2)
Income–*No*. *(%)*					
	$0 - $49,000	131 (36)	51 (43)	48 (39)	32 (27)
	$50,000 to $99,000	125 (35)	41 (34)	42 (34)	42 (35)
	$100,000 or more	87 (24)	24 (20)	27 (22)	36 (30)
	Prefer not to say	18 (5)	4 (3)	5 (4)	9 (8)
Residence–*No*. *(%)*					
	Rural	77 (21)	23 (19)	30 (25)	24 (20.2)
	Small (less than 100,000)	58 (16)	15 (13)	17 (14)	26 (21.8)
	Suburban near large city	157 (44)	54 (45)	55 (45)	48 (40.3)
	Mid-sized city (100,000 to 1million)	29 (8)	11 (9)	8 (7)	10 (8.4)
	large city more than 1 million	40 (11)	17 (14)	12 (10)	11 (9.2)

**Fig 2:**
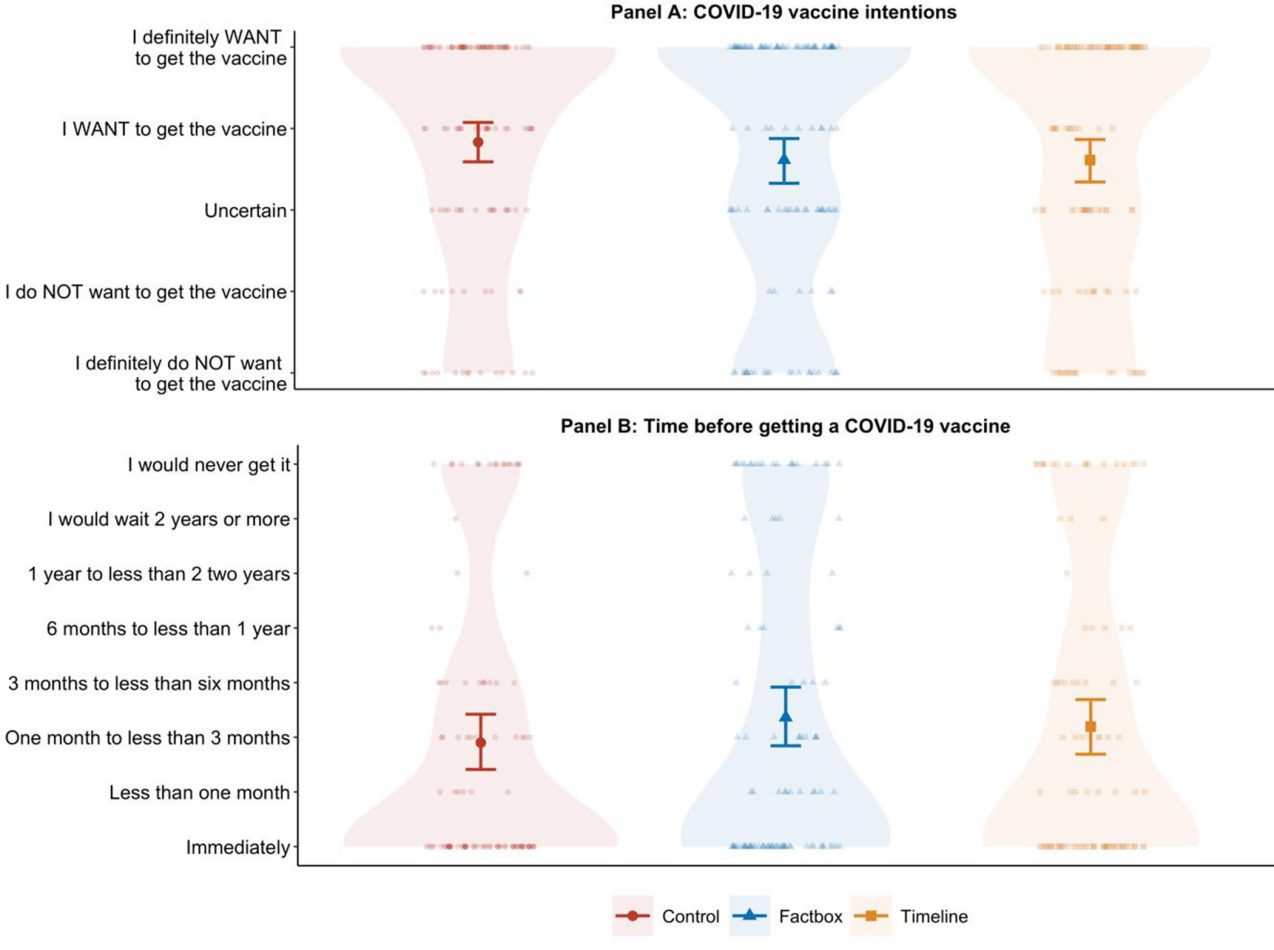
Mean COVID-19 vaccine intention scores for the factbox message (showing risk of getting COVID-19 versus risk of getting a COVID-19 vaccine), the timeline message (showing the development of mRNA and COVID-19 vaccine research), and control group. The middle bold line represents the mean and the error bars represent 95% confidence intervals. Individual data points are displayed along with shaded density distributions.

We found no statistically significant differences between groups regarding vaccination intentions, *F*(2,358) = 0.86, *p* = .425, $\eta_{P}^{2}$ = .005, or the time respondents would wait to get vaccinated, *F*(2,306) = 0.79, *p* = .453, $\eta_{P}^{2}$ = .005. In fact, as shown in Fig 2 and Table 2, although there were no statistically significant differences between groups, mean scores for both the intervention groups were slightly lower for vaccine intentions (i.e., *less* willing to get a COVID-19 vaccine) and slightly higher for the time respondents would wait to get vaccinated (i.e., would want to wait *longer*) as compared to those in the control group who did not see any message.

**Table 2:** Outcome measures according to group assignment.

	Control	Factbox	Timeline	Control	Control	Factbox
	(N = 120)	(N = 122)	(N = 119)	vs. Factbox	vs. Timeline	vs. Timeline
	*Means*±*SD*			*Mean differences (95%CIs)*		
Vaccine intentions *(Scale range*: *1–5)*	3.83±1.40	3.61±1.56	3.61±1.53	0.22 (-0.16 to 0.59)	0.22 (-0.15 to 0.59)	0.00 (-0.39 to 0.39)
Time to vaccine *(Scale range*: *1–8)*	2.90±2.58	3.36±2.77	3.20±2.70	-0.46 (-1.19 to 0.26)	-0.29 (-1.03 to 0.45)	0.17 (-0.58 to 0.92)
Vaccine safety *(Scale range*: *1–5)*	3.57±1.38	3.52±1.43	3.45±1.33	0.04 (-0.31 to 0.40)	0.11 (-0.23 to 0.46)	0.07 (-0.28 to 0.42)
Vaccine side effects *(Scale range*: *1–5)*	2.66±1.50	2.52±1.38	2.87±1.42	0.14 (-0.23 to 0.51)	-0.22 (-0.59 to 0.16)	-0.36 (-0.71 to 0.00)

These results did not differ when controlling for respondents’ age, (no statistically significant effect of group on vaccination intentions, *F*(2,355) = 0.06, *p* = .945, $\eta_{P}^{2}$ < .001, and no statistically significant effect of group on time respondents would wait to get vaccinated, *F*(2,303) = 0.05, *p* = .950, $\eta_{P}^{2}$ < .001), or gender, (no statistically significant effect of group on vaccination intentions, *F*(2,352) = 1.46, *p* = .233, $\eta_{P}^{2}$ = .008, and no statistically significant effect of group on time respondents would wait to get vaccinated, *F*(2,301) = 1.54, *p* = .217, $\eta_{P}^{2}$ = .010). Furthermore, we found no statistically significant difference on respondents’ views about how safe COVID-19 vaccines are, *F*(2,358) = 0.20, *p* = .815, $\eta_{P}^{2}$ = .001, and worry about side effects, *F*(2,357) = 1.89, *p* = .152, $\eta_{P}^{2}$ = .010, between the three groups.

### Exploratory results

We found that results from the subset of respondents who spent at least 10 seconds on the page displaying the intervention messages (n = 298) did not differ from the overall sample: there were no statistically significant differences in vaccination intentions, *F*(2,295) = 1.51, *p* = .224, $\eta_{P}^{2}$ = .010, and no significant difference in time respondents would wait to get vaccinated between groups, *F*(2,252) = 1.08, *p* = .342, $\eta_{P}^{2}$ = .008.

For the subset of respondents who had previously reported that they were hesitant about getting a COVID-19 vaccine (in an earlier survey fielded in December 2020; n = 86), we also found no statistically significant differences between groups on respondents’ vaccination intentions, *F*(2,83) = 0.32, *p* = .724, $\eta_{P}^{2}$ = .008, or the time they would wait to get vaccinated, *F*(2,61) = 1.05, *p* = .356, $\eta_{P}^{2}$ = .033.

**Fig 3:**
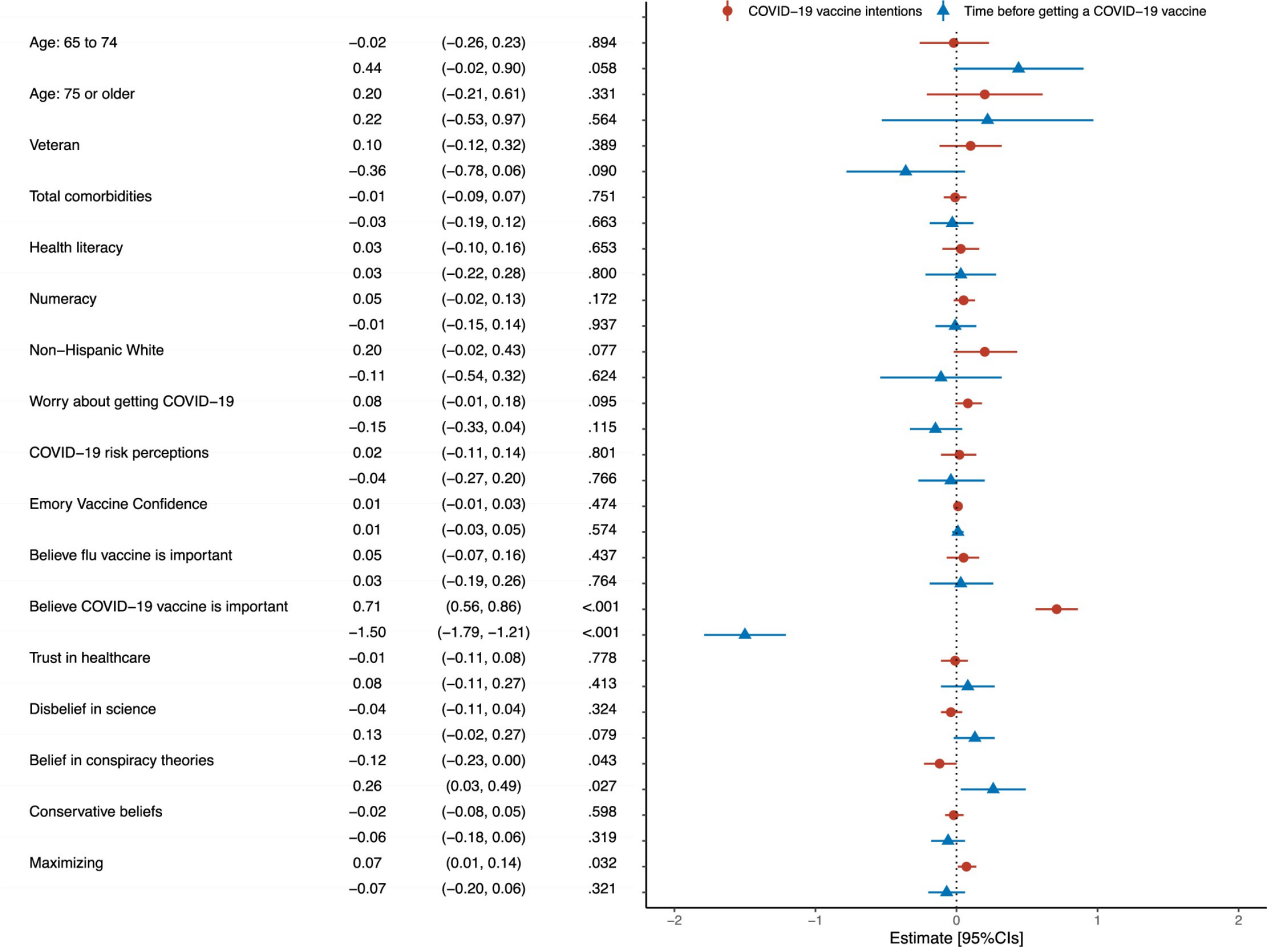
Predictors of respondents’ COVID-19 vaccine intentions and how long they would wait before getting a COVID-19 vaccine. Model estimates are represented by the triangular and circular point shapes with error bars for the 95% confidence intervals. Reference groups are: age: ≤64, non-Veteran, and any other Race/Ethnicity. R^2^ adjusted: Vaccine intentions = 0.68, Time to vaccine = 0.70.

As shown in Fig 3, believing that it is important for adults to get the COVID-19 vaccine predicted greater intentions to get a COVID-19 vaccine (0.71, 95%CI[0.56 to 0.86], *p* < .001) and to get one sooner, (-1.50, 95%CI[-1.79 to -1.21], *p* < .001). Conversely, belief in COVID-19 related conspiracy theories predicted lower intentions to get a COVID-19 vaccine (-0.12, 95%CI[-0.23 to 0.00], *p* = .043) and wanting to wait longer before getting one (0.26, 95%CI[0.03 to 0.49], *p* = .027). Preferring to taking action in situations where it is not clear whether or not medical action is needed also predicted greater COVID-19 vaccine intentions (0.07, 95%CI[0.01 to 0.14], *p* = .032).

## Discussion

Major advances in vaccine development are at risk of being undermined by increasing levels of vaccine hesitancy [42]. In the immediate term, public hesitancy towards COVID-19 vaccines presents a notable barrier to limiting the spread of COVID-19 [2]. Messaging designed to address prominent concerns about COVID-19 vaccines might encourage people who are otherwise hesitant about getting vaccinated to receive one.

In the present study, we found no evidence that neither a message comparing the potential risks of getting COVID-19 to the potential risks associated with COVID-19 vaccines nor a timeline used to communicate the vaccine development process, had any effect on respondents’ intentions to get a COVID-19 vaccine, the time that they would want to wait before getting vaccinated, perceived safety of COVID-19 vaccines, or worry about vaccine-related side effects. These results were unanticipated and remained consistent when controlling for the age and gender of respondents and following exploratory analyses of respondents who spent at least 10 seconds looking at the messages and among those who had previously expressed that they did not want to get a COVID-19 vaccine.

The messages were designed to address concerns about the risks from COVID-19 vaccines and the vaccine development process as they are reasons frequently cited by people who are hesitant about receiving a COVID-19 vaccine as we found in a previous survey of these same participants [27] as well as in the literature more generally [14, 17]. The absence of an intervention effect is unfortunate as effective low-cost messaging strategies could contribute substantially to combatting growing levels of vaccine hesitancy and help reduce the impact of the COVID-19 pandemic.

One potential reason for these null findings could simply be that the information in the two messages was not sufficiently convincing to influence the vaccine intentions of people who are concerned about the risks of vaccines and the development process. For individuals who did not have concerns about the development or safety of COVID-19 vaccines, it could also have been the case that the information in the messages may have drawn their attention to these issues which may have then served to undermine their prior confidence in vaccine development and safety [43]. It is unlikely that these null effects were due to insufficient statistical power. Observed effect sizes in this experiment were very small (such that even if significant, the meaningfulness of these differences would have been questionable), and if anything, the intervention groups showed slightly more vaccine hesitancy than the control group, not less.

Given the cross-sectional nature of this experiment, it is not possible to examine whether repeated exposure to these messages may have yielded more positive results. Repeated messaging about health issues is often more effective at changing attitudes and behaviors [44], however, given the prevalence of media coverage about COVID-19 and vaccine development it is also possible that efficacy of these messages may be suppressed by respondents having high prior exposure to similar information. In addition, while many studies have shown changes in respondents’ vaccine intentions directly after seeing information about COVID-19 vaccines [32, 45–50] it could be the case that an effect of the provided information on respondents’ intentions may only emerge over a longer period than was covered in the study. Another possibility, given how public attitudes and intentions towards the COVID-19 vaccines have varied over time [51], is that we may have observed different results if the study had been conducted at another time during the pandemic, or with a longer follow up period.

The design of the message–regarding both the style and content, may have also contributed to the null finding. However, given the data available from the present study it is not possible to posit informed explanations for the null findings based on respondents’ perceptions of the messages themselves. This does reveal an important avenue for future research, which might leverage qualitative methods (e.g., focus groups or open responses) in order to better understand how people perceived the messages used in the present study.

The present findings are aligned with research which suggests that providing corrective information based on an information-deficit assumption is not always effective at addressing people’s concerns and doubts [52–54] and highlight the need for testing public health messages to ensure they are as effective as possible as well as to identify potentially counterproductive effects they may have. Future research might explore whether different message styles or sources (e.g., non-partisan experts or popular figures) may be more effective at sufficiently addressing concerns about vaccine development and safety. The results of the exploratory regression analyses suggested that the most influential predictor of respondents’ COVID-19 vaccine intentions was their general beliefs about the importance of COVID-19 vaccines for adults. Our findings suggest that negative views about the importance and need for COVID-19 vaccines are strongly held beliefs which are difficult to address using informative health messages. However, as a number of studies have reported small benefits in vaccine intentions following communications about COVID-19 vaccine side effects and safety development concerns [32, 49], health messages aiming to reassure people about the importance of COVID-19 vaccines and focusing on the benefits remain an important strategy for rapid and widespread communication of this critical health information [46–48].

Identifying methods for addressing complex and deeply held beliefs in COVID-19 related conspiracy theories, which we found predicted lower vaccine intentions, remains a key goal for public health communication research. As respondents’ preferences for taking medical action instead of watching and waiting in cases where it is unclear which option is needed predicted vaccine intentions, communications which focus on the active nature of “getting a vaccine” may resonate with these individuals. Thus, we believe these findings are important in highlighting the need for research into communications that address other influences of vaccine intentions that might be more receptive to this type of health messaging strategy and to identify alternative methods for addressing concerns about safety and development.

### Limitations

Despite evidence that self-reports are good predictors of health behaviors [55], it is important that these findings are considered in the context of known limitations of this method (e.g., social desirability in respondents’ answers). The study design (i.e., US-based recruitment, online, and in English) prevents generalization of the present findings outside of the US and to people who may have limited internet access and lower English proficiency. Furthermore, our sample consists of both Veteran and non-Veteran respondents completing the third (and final) survey of a longitudinal study, which began in December, 2020 and thus are not representative of the general population.

## Conclusion

In the present study, providing corrective information about the risks of getting COVID-19 compared to receiving a COVID-19 vaccine and about the development of COVID-19 vaccines was not effective at promoting intentions to get a COVID-19 vaccine. Further research is needed to identify how to effectively address concerns about the risks of side effects from COVID-19 vaccines and the vaccine development process.
